# Photoplethysmography Driven Hypertension Identification: A Pilot Study

**DOI:** 10.3390/s23063359

**Published:** 2023-03-22

**Authors:** Liangwen Yan, Mingsen Wei, Sijung Hu, Bo Sheng

**Affiliations:** 1 School of Mechatronic Engineering and Automation, Shanghai University, Shanghai 200444, China; 2 School of Electronic, Electrical and Systems Engineering, Loughborough University, Ashby Road, Loughborough, Leicestershire LE11 3TU, UK

**Keywords:** hypertension, photoplethysmography, Long Short-Term Memory, attention mechanism

## Abstract

To prevent and diagnose hypertension early, there has been a growing demand to identify its states that align with patients. This pilot study aims to research how a non-invasive method using photoplethysmographic (PPG) signals works together with deep learning algorithms. A portable PPG acquisition device (Max30101 photonic sensor) was utilized to (1) capture PPG signals and (2) wirelessly transmit data sets. In contrast to traditional feature engineering machine learning classification schemes, this study preprocessed raw data and applied a deep learning algorithm (LSTM-Attention) directly to extract deeper correlations between these raw datasets. The Long Short-Term Memory (LSTM) model underlying a gate mechanism and memory unit enables it to handle long sequence data more effectively, avoiding gradient disappearance and possessing the ability to solve long-term dependencies. To enhance the correlation between distant sampling points, an attention mechanism was introduced to capture more data change features than a separate LSTM model. A protocol with 15 healthy volunteers and 15 hypertension patients was implemented to obtain these datasets. The processed result demonstrates that the proposed model could present satisfactory performance (accuracy: 0.991; precision: 0.989; recall: 0.993; F1-score: 0.991). The model we proposed also demonstrated superior performance compared to related studies. The outcome indicates the proposed method could effectively diagnose and identify hypertension; thus, a paradigm to cost-effectively screen hypertension could rapidly be established using wearable smart devices.

## 1. Introduction

In recent years, the incidence and mortality rates of cardiovascular disease worldwide have continued to rise, with the age of onset advancing [1]. By 2020, the total number of cardiovascular disease patients in China had reached 330 million, and the out-of-hospital mortality rate was significantly higher than the in-hospital mortality rate [2]. Hypertension, an independent risk factor for cardiovascular and cerebrovascular diseases, has a substantial impact on cardiovascular health [3]. Research has shown that there are 245 million hypertensive patients in the Chinese population aged 18 and above. The total cardiovascular and cerebrovascular event risk of the population with normal high blood pressure increased by 37.0%, while the risk of ischemic stroke increased by 56.0%. The “Chinese Clinical Practice Guidelines for Hypertension” have also lowered the diagnostic threshold for hypertension by 10 mmHg, aimed at enabling timely treatment for patients. Early detection of hidden hypertension risks has significant implications for preventing cardiovascular and cerebrovascular diseases.

Blood pressure refers to the lateral pressure that flowing blood in blood vessels exerts on the unit area of the blood vessel wall. It is generally divided into two types: SBP (Systolic Blood Pressure) and DBP (Diastolic Blood Pressure) [4], measured in millimeters of mercury. While arterial intubation is the international gold standard for blood pressure detection [5], this technology requires invasive procedures and is unsuitable for daily blood pressure measurement. Currently, mercury or electronic sphygmomanometers are commonly used for blood pressure measurement in homes and communities [6], but there are several limitations to these devices, such as the need for special instruments, cumbersome operation, inconvenient portability, and high equipment costs. These factors may discourage patients from continuing blood pressure detection, even though regular monitoring is crucial in helping patients better manage blood pressure and avoid serious cardiovascular complications [7]. People with hypertension are more susceptible to emotional problems, such as clinical depression [8,9], anxiety, and greater stress [10], which can contribute to changes in blood pressure [11]. However, traditional blood pressure monitoring methods do not capture these risks. In recent decades, non-invasive, continuous, and sleeveless alternatives for assessing blood pressure have gained increasing attention, most of which are based on the analysis of physiological signals. Researchers have explored different methods to evaluate blood pressure, such as calculating PTT (pulse pave transmit time) using ECG (electrocardiograph) and PPG (photoplethysmography) signals [12] and using simultaneous measurements of PPG from the toes and fingertips to determine PTT [13]. These methods require the synchronous acquisition of two sensors, and the measurement process is complex. Therefore, blood pressure detection based on a single PPG signal has become a new research focus. Many researchers have used machine learning to extract relevant features from PPG signals to estimate blood pressure. For instance, Monte-Moreno [14] extracted time-domain and frequency-domain features from PPG waveforms and estimated blood pressure using a random forest algorithm. Nour et al. [15] adopted four machine learning methods to classify PPG signals in hypertensive patients and concluded that the best methods for classifying hypertension types were the Decision Tree and Random Forest classifiers. Although these artificial feature extraction methods are highly subjective, the PPG signal can drift or distort under the influence of illumination, motion, or equipment, making feature extraction challenging and less accurate [16]. Deep learning methods can extract more abstract and complex features from input signals. For instance, Tjahjadi et al. [17] extracted time-domain information and used BiLSTM to classify blood pressure with a remarkable 97.33% accuracy rate for hypertension. Liang et al. [18] converted PPG signals into RGB images and applied convolutional neural networks to evaluate hypertension, achieving F1-scores of 80.52%, 92.55%, and 82.95% for normal blood pressure, prehypertension, and hypertension, respectively. Wu et al. [19] employed continuous wavelet transform to convert the original PPG signal into a pixel scalogram, and then trained and validated their CNN model with a 90% accuracy rate. However, the feature extraction and deep learning approach may not fully exploit the advantages of neural networks, and the method of converting a one-dimensional PPG signal into an image may increase the signal dimension and prolong the model training time. Given that PPG signals are periodic and non-stationary, it is crucial to enhance the attention and connection between the signals and the early-stage signals for long-term blood pressure detection and evaluation.

The study aims to explore a hypertension recognition method by the means of PPG signals to provide an alternative and cost-effective clinical monitoring tool. Thus, a bespoke data acquisition system has been developed to collect PPG signals. Furthermore, a Philips DB12 dataset as a reference was hired to evaluate the performance and accuracy of the bespoke data acquisition device. A model named LSTM for hypertension identification based on the attention mechanism was established to use raw data as input to avoid the tedious process of feature extraction. To verify and evaluate the performance, the performance of different models on the same dataset was applied to compare their metrics such as accuracy, precision, recall, F1-score, and operating efficiency.

## 2. Materials and Methods

### 2.1. Bespoke Data Acquisition Device and Its System

**Figure 1:**
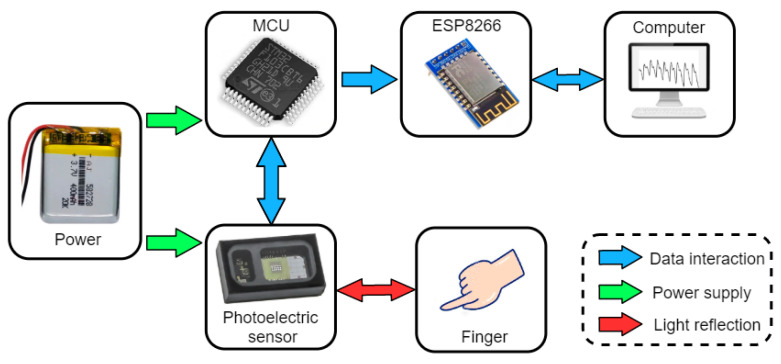
A flowchart of the PPG data acquisition system.

To obtain high-quality raw PPG signals, we have developed a complete PPG signal acquisition system, as illustrated in Figure 1. The system is mainly divided into four modules: the PPG photoelectric sensor module, power supply module, control module, and communication module.

**Figure 2:**
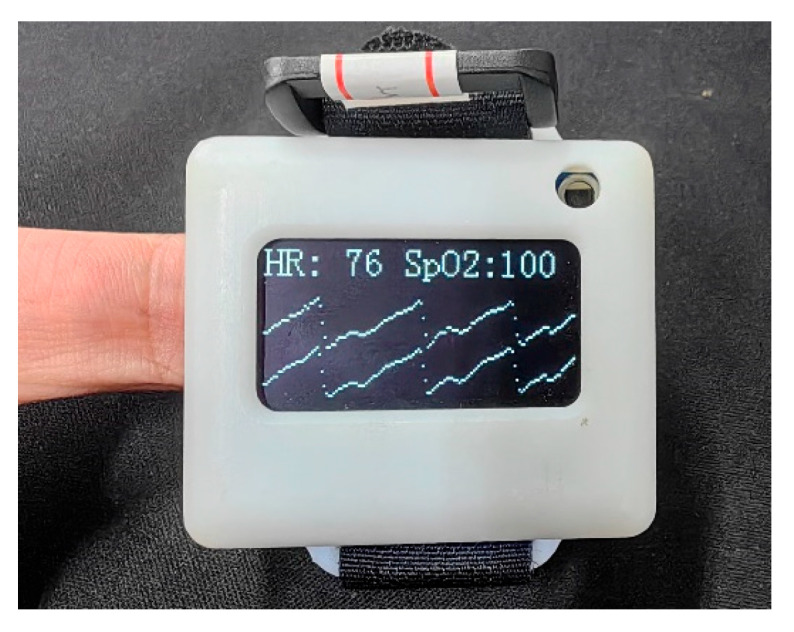
PPG data acquisition device.

The main objective of this system is to acquire, transmit, display, and store PPG signals. The photoelectric sensor, as the primary data source, has a direct impact on the accuracy and efficiency of subsequent data processing. In this study, we used the MAX30101 reflective photoelectric pulse sensor manufactured by Maxim Integrated (San Jose, CA, USA). This sensor has three LEDs, an adjustable constant current source drive, and a programmable sampling frequency. Additionally, the module is designed with low power consumption and high output data capacity, which can efficiently meet various sampling requirements. For data transmission, we used an ATK-ESP8266 as the Wi-Fi module, which supports standard IEEE802.11b/g/n protocols and the TCP/IP protocol stack and provides complete Wi-Fi functionality. The microcontroller selected for the system is the high-performance 32-bit MCU-STM32F103C8T6, designed with an ARM Cortex-M3 core. The CPU frequency of this microcontroller can reach up to 72 MHz, and its performance and chip resources are sufficient for PPG signal collection and forwarding. Figure 2 shows the PPG acquisition equipment developed in this study, and Table 1 summarizes its performance.

To confirm the performance and precision of the experimental equipment, the heart rate measurement displayed by the PHILIPS DB12 blood oxygen monitor (Amsterdam, The Netherlands) was utilized as a benchmark for comparison before experimenting, as presented in Table 2. The heart rate data that was calculated from the PPG signal that was collected and processed had an error margin of less than 2% when compared to the heart rate that was recorded by the DB12 blood oxygen monitor, thus guaranteeing the precision of the data acquisition.

### 2.2. Data Acquisition

The participants were divided into two groups: the hypertension group and the healthy group. The hypertension group comprised 15 campus faculty and staff members who were diagnosed with hypertension. Similarly, 15 faculty and staff members without hypertension on campus were selected as the healthy group during the same period. In total, there were 30 subjects, consisting of 16 males and 14 females, with an age range of 50 ± 10 years.

**Figure 3:**
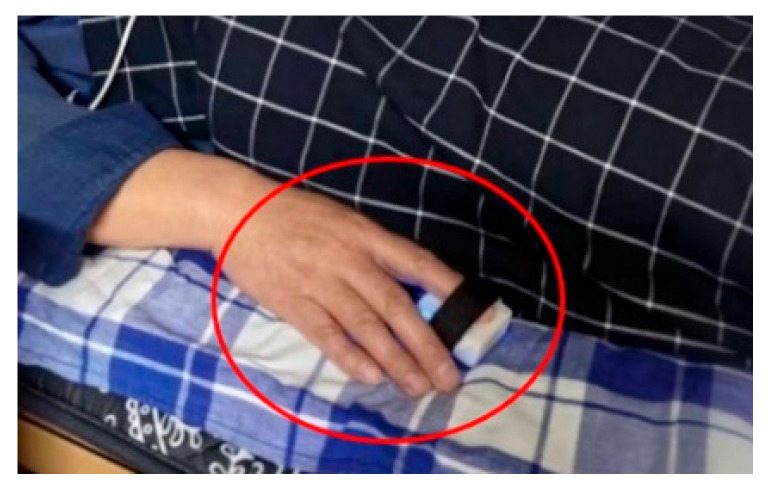
PPG signal collection experiment.

The basic information of all volunteers, including age, gender, BMI (Body Mass Index), and SBP, are presented in Table 3. Meanwhile, the PPG signals of the patients were collected in a controlled environment where the temperature of the room was set to 23 °C, and the finger temperature was maintained at 32 °C. The participants were instructed to remain still while their right index finger PPG signal was collected for 30 min, with one collection in the morning, one in the afternoon, and one in the evening, resulting in three samples per person, each with a sampling frequency of 100 Hz. The data collection process is depicted in Figure 3.

Diagnostic criteria: volunteers with systolic blood pressure ≥ 140 mmHg and/or diastolic blood pressure ≥ 90 mmHg (1 mmHg = 0.133 kPa) or those with a confirmed diagnosis of hypertension and were then taking hypertension medication were included in the hypertension group.

Exclusion criteria: various acute, infectious, and contagious diseases; individuals with impaired visceral function; and those with moderate or above stenosis of the limb arteries were excluded from the study. The protocol for this study strictly adheres to the Helsinki Declaration [20], and the volunteers who agreed to participate in this study signed an informed consent form and obtained approval through the ethics committee of Shanghai University.

### 2.3. Data Preprocessing

**Figure 4:**
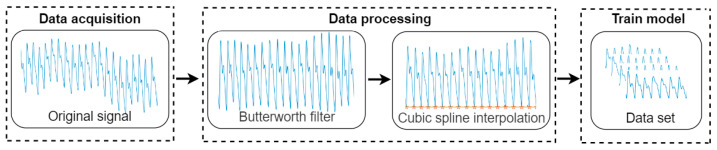
Data processing flow chart.

PPG is a non-stationary random signal that is characterized by low amplitude, low frequency, and susceptibility to interference. The quality of pulse signal acquisition can be affected by noise interference, mainly including baseline drift and high-frequency noise [21]. Baseline drift can be caused by respiration and body movement during signal acquisition, which leads to distortion of the PPG signal waveform. Therefore, data preprocessing is required before model training. In this study, the collected PPG raw signal was first filtered using a Butterworth digital filter to remove noise interference, followed by baseline drift removal using cubic spline interpolation. The data preprocessing flowchart is shown in Figure 4.

#### 2.3.1. Butterworth Filter

The Butterworth filter is an electronic filter characterized by a maximally flat frequency response curve within the passband, without any ripples, and gradually decreasing to zero within the stopband. On the Bode plot of amplitude versus logarithmic angular frequency, the amplitude decreases gradually with increasing frequency, tending towards negative infinity, starting from a certain boundary frequency [22]. The squared magnitude response of the Butterworth filter is expressed by Equation (1):

(1) $${\left|H\left(j\omega \right)\right|}^{2}=\frac{1}{1+{\left(\frac{\omega }{\omega_{c}}\right)}^{2N}}$$

*N* represents the filter order. The larger the *N*, the greater the approximation of the passband and stopband, and the steeper the transition. $\omega_{c}$ is the cutoff frequency of the filter.

The pulse wave frequency of healthy adults typically ranged from 0.1–20 Hz, with most of the invalid energy concentrated near the DC component. This energy accounted for approximately 95% of the total energy of the waveform and reflected the characteristics of the heart pressure wave [23]. Meanwhile, the respiratory frequency of healthy adults was generally around 0.15–0.5 Hz. Therefore, the cut-off frequency of the filter was set at 0.6 Hz, and the second-order Butterworth filter was adopted to eliminate low-frequency noise caused by various factors, including baseline drift from different finger pressure and respiration. Finally, we smoothed the original signal using the smooth function.

**Figure 5:**
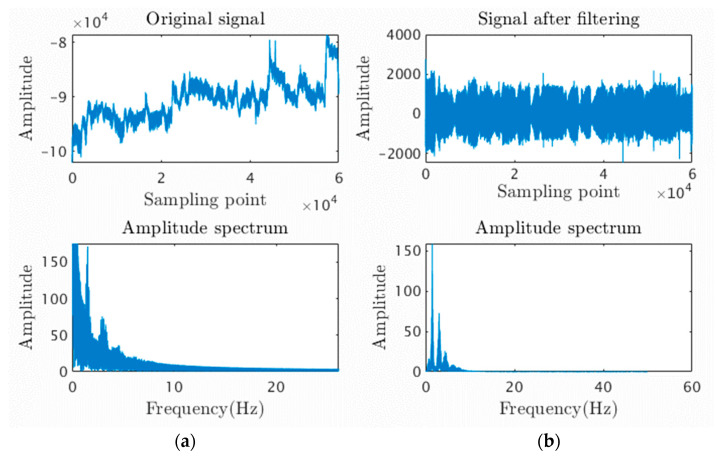
(**a**) Original signal and its spectrum; (**b**) Signal and spectrum after filtering.

As shown in Figure 5, most of the baseline drift was removed by the Butterworth digital filter, and the distribution of waveform is more concentrated near the central axis. The amplitude spectrum diagram shows that the PPG frequency distribution is more focused within 10 Hz, with three distinct frequency peaks, indicating the filtering out of low-frequency noise. This processing successfully restores the original human PPG waveform, providing a solid foundation for the subsequent feature extraction of PPG.

#### 2.3.2. Cubic Spline Interpolation

To better extract accurate feature information, this study uses cubic spline interpolation to remove residual offset components in the signal processed, as mentioned earlier [24]. Cubic spline interpolation uses a smooth curve that can pass through a predetermined fixed point. The basic principle is to connect the points between each other using a cubic curve $P_{i}\left(x\right)$, and $P_{i}\left(x\right)$, $P_{i}^{\prime }\left(x\right)$, and $P_{i}^{\prime \prime }\left(x\right)$ are continuous at each boundary point. Because the second derivatives are continuous at all points, adjacent piecewise cubic polynomials have better coupling and a uniform curvature, making the curve smoother. The definition equation of the cubic spline can be expressed by Equation (2).

(2) $$P_{i}\left(\hat{x}\right)=a_{i}+b_{i}{\left(\hat{x}-x_{i}\right)}^{\;}+c_{i}{\left(\hat{x}-x_{i}\right)}^{2}+d_{i}{\left(\hat{x}-x_{i}\right)}^{3}$$

**Figure 6:**
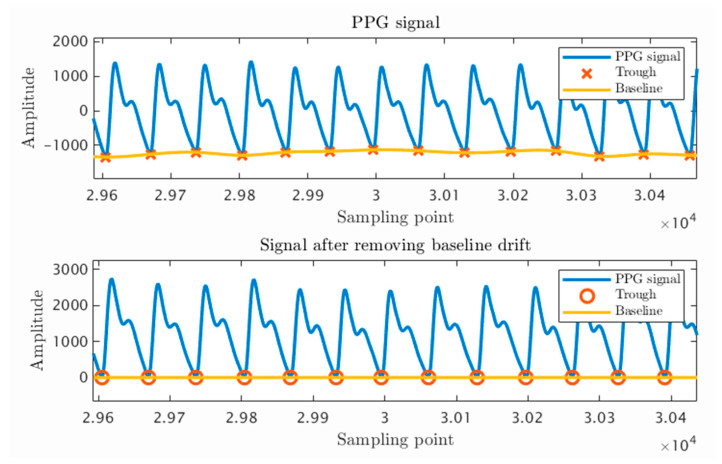
Cubic spline difference removes the baseline deviation of the PPG trough.

The baseline drift of the PPG signal is mainly caused by the fact that the trough positions of each cycle are mostly different from the same baseline. Therefore, a fitting curve can be constructed by taking the corresponding positions of trough points of each two cycles as nodes and fitting them with a spline function. By subtracting the fitting curve sequence from the PPG signal sequence, an un-offset PPG signal can be obtained. As shown in Figure 6, the curve fitted by cubic spline interpolation can accurately represent the current waveform offset, and the troughs of the PPG waveform after removing the offset are all placed at the same height. This establishes the benchmark for feature extraction and selection. It is only after eliminating noise and offset that relevant physiological information in the human body can be accurately displayed, which is also important for cleaning datasets in machine learning and deep learning. This is a noteworthy task in the model training process.

### 2.4. LSTM-Attention Model

This section mainly introduces the LSTM-Attention model in four parts. The first part is the structure of the LSTM-Attention model. The second part describes the specific parameters of each network layer. The third part is the training of the model. The fourth part is the evaluation metrics of the model.

#### 2.4.1. Model Architecture

**Figure 7:**
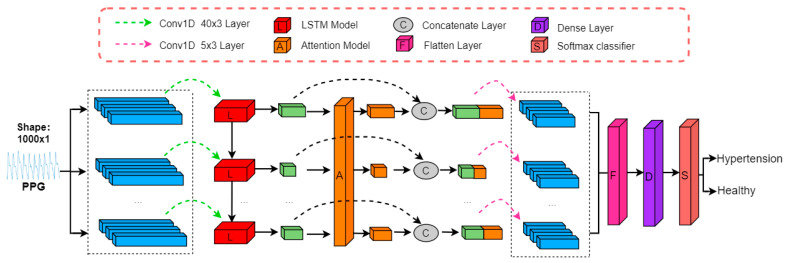
Structure diagram of LSTM-Attention.

The proposed model comprises convolutional layers, LSTM network layers, and attention layers. It features one input node in the input layer and predicts a sequence of the next 1000-time steps in the output layer, taking filtered and denoised PPG signals as input. To extract deeper PPG features, the model employs three convolutional kernels with a stride of 40 in the first convolutional layer, expanding the feature dimension. Next, it maps the expanded features to 5 LSTM modules to generate feature maps. LSTM allows associating information from previous and subsequent time steps, enabling the model to predict the output at the next time step based on the previous input, thus anticipating future changes. Then, the information processed by the LSTM module feeds into the attention mechanism module. This module analyses the signal’s key and non-key regions and assigns different weights to signals in different regions, placing greater emphasis on details that require attention and enhancing data analysis accuracy. Finally, the model integrates feature information through the second convolutional kernel, reduces data dimensionality through the Flatten layer, conducts full connection via the Dense layer, and obtains the classification result through the Softmax function. The specific network structure is illustrated in Figure 7.

##### LSTM Block

Deep learning leverages hierarchical network structures to perform high-level abstraction on data. By learning from vast amounts of data, it extracts key features and computes nonlinear patterns. In the hypertension recognition model, the deep learning algorithm learns from the input pulse wave data, automatically extracting features from it. By avoiding the need for manual feature extraction for specific feature types, the model can resist interference and generalize better.

**Figure 8:**
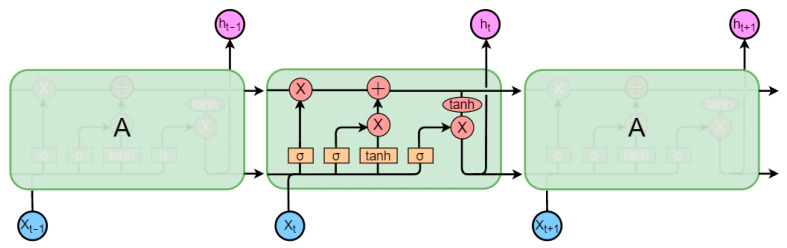
Schematical diagram of LSTM.

Pulse wave data is a type of one-dimensional time-series data, and deep learning algorithms have shown promising results in processing such data [25]. Time-series data points are related to each other across different time points, and RNN (Recurrent Neural Network) structures are designed to capture such correlations and extract relevant features. RNN connects the previous and current nodes of the input data, allowing the calculation of the current node to depend on both the current input and the output of the previous time step. This approach enables the network to capture nonlinear relationships that match the time-series data pattern. However, RNN suffers from a long-term dependency problem, where the memory of the neuron for the input information at earlier time steps decreases as the network depth increases, leading to the significant influence of the final input [26]. This problem can cause gradient disappearance or explosion during the optimization stage. Therefore, in this experiment, we use the LSTM network structure to optimize the standard RNN model. Figure 8 illustrates the schematic diagram of the LSTM structure.

The LSTM architecture effectively addresses the issues of gradient vanishing and exploding that often arise when processing time-series data with traditional RNNs. In contrast to the neural structure of traditional RNNs, LSTM incorporates three “gates” [27] that optimize its architecture. The “gate” structure comprises a Sigmoid operation, a sum operation, and a multiplication operation, with the output of the Sigmoid function ranging between 0 and 1. This output can be thought of as the amount of output information obtained after processing the input information, leading to the metaphorical reference of a “gate” structure. This “gate” structure enables neural nodes to selectively process the received information, thereby screening out key features.

In LSTM, there are three types of gates: the forget gate, the input gate, and the output gate. The forget gate receives input from the current time step $x_{t}$, the previous output $h_{t-1}$, and the previous cell state $C_{t-1}$. The forget gate selectively “forgets” some of the information in the previous cell state $C_{t-1}$, based on the current input.

The activation Equation for the forget gate (where b and W are value vectors, and σ is the sigmoid function) is:

(3) $$f_{t}=\sigma \left(W_{f}\times \left[x_{t},h_{t-1},C_{t-1}\right]+b_{f}\right)\times C_{t-1}$$

After discarding irrelevant information through the forget gate, the neuron node uses the input gate to obtain new information at this time and update its state $C_{t}$ based on $x_{t}$, $C_{t-1}$, $h_{t-1}$, and the activation function tanh.

(4) $$i_{t}=\sigma \left(W_{i}\times \left[x_{t},h_{t-1},C_{t-1}\right]+b_{i}\right)$$

(5) $$C_{t}=f_{t}+i_{t}\times \tanh\left(W_{c}\times \left[x_{t},h_{t-1},C_{t-1}\right]+b_{c}\right)$$

Based on the new state $C_{t}$ of the neuron, the LSTM structure can obtain the output of the current neuron through the output gate according to $h_{t-1}$, $x_{t}$, and the new state $C_{t}$.

(6) $$o_{t}=\sigma \left(W_{o}\times \left[x_{t},h_{t-1},C_{t}\right]+b_{o}\right)$$

(7) $$h_{t}=\tanh\left(C_{t}\right)\times o_{t}$$

The output of a recurrent neural network can take different forms such as the corresponding judgment result for each input at every time step, a prediction of the next data point based on the learned temporal patterns, or a holistic judgment of the entire input sequence. For hypertension recognition in this study, the LSTM network is used to identify hypertension in a given data segment, and the output of the network represents a judgment of whether the data segment is likely to be in a hypertensive state.

##### Attention Block

**Figure 9:**
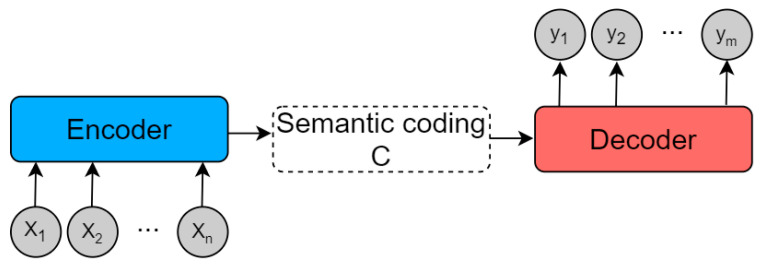
Abstract Encoder-Decoder framework.

The attention mechanism, proposed by Bahdanau in 2014 [28], mimics the image-processing mechanism unique to the human brain. This method selectively allocates attention according to needs, improving the efficiency of information processing in computers. Attention mechanisms are now widely used in various deep-learning fields, such as machine translation [29], image processing [30], and text classification [31], among others. Typically, attention mechanisms are based on the Encoder–Decoder framework, as shown in Figure 9.

**Figure 10:**
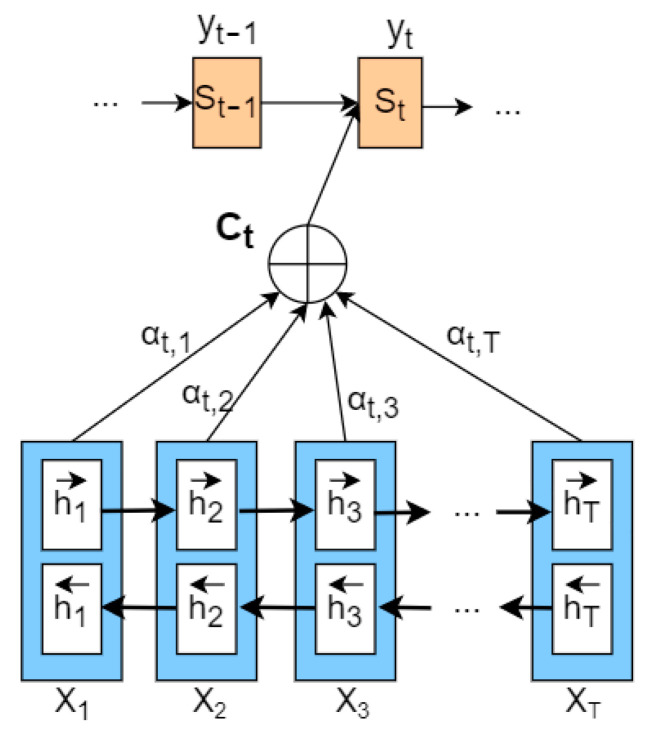
Schematic diagram of attention mechanism structure.

The model maps a variable-length input $X=\left(x_{1},x_{2},\ldots ,x_{n}\right)$ to a variable-length output $Y=\left(y_{1},y_{2},\ldots ,y_{m}\right)$. The Encoder transforms the variable-length input sequence *X* into an intermediate semantic representation *C* via a non-linear transformation: $C=f\left(x_{1},x_{2},\ldots ,x_{n}\right)$. The Decoder’s task is to predict and generate the output $y_{i}$ at the time *i*, based on the intermediate semantic representation *C* and the previously generated $y_{1},y_{2},\ldots ,y_{i-1}$:$y_{i}=g\left(y_{1},y_{2},\ldots ,y_{i-1},\;C\right)$, where f() and g() are both non-linear transformation functions. The attention mechanism proposed by Bahdanau et al. can address the issue of the lack of discriminability in the input sequence *X* for traditional Encoder–Decoder frameworks. The model framework is shown in Figure 10.

$S_{t-1}$ is the hidden state of the decoder at time t − 1, $y_{t}$ is the target value, $C_{t}$ is the context vector, and then the hidden state at time t is given by Equation (8).

(8) $$S_{t}=f\left(S_{t-1},y_{t-1},C_{t}\right)$$

$C_{t}$ depends on the hidden layer representation of the input sequence on the encoding side and can be represented, as shown in Equation (9), through weighted processing.

(9) $$C_{t}=\overset{T}{\underset{j=1}{\sum }}\alpha_{t,j}h_{j}$$

$h_{j}$ refers to the hidden vector of the jth value on the encoder side. It contains information about the entire input sequence but focuses on the surrounding parts of the jth value. *T* is the length of the input side. $\alpha_{t,j}$ represents the attention allocation coefficient of the jth value on the encoder side to the tth value on the decoder side, and the sum of the probability values of $\alpha_{t,j}$ is 1. The calculation formula for $\alpha_{t,j}$ is shown in Equations (10) and (11).

(10) $$\alpha_{t,j}=\frac{\exp\left(\alpha_{t,j}\right)}{\sum_{j=1}^{T}\exp\left(\alpha_{t,j}\right)}\;$$

(11) $$\alpha_{t,j}=a\left(S_{t-1},h_{j}\right)$$

$\alpha_{t,j}$ represents an alignment model that measures the alignment/influence between the value at position j in the Encoder and the value at position t in the Decoder. Typically, the alignment model is parameterized as a feedforward neural network and trained jointly with the rest of the system.

In the hypertension recognition experiment, local spatial features of the photoplethysmography signal were obtained through convolutional layers, and temporal feature representations of the PPG signal were further obtained through the LSTM network. However, these components did not effectively capture the correlation between current and past/future sampling points of the PPG signal, which can be detrimental for long-term PPG signal recognition tasks. To address this issue and improve the correlation between distant sampling points, an attention mechanism was introduced in this experiment. This mechanism selectively allocates different attention resources to sampling points with varying levels of importance. By incorporating the attention mechanism into the model, the spatial and temporal features of the PPG signal can be obtained, and the correlation between the current sampling point and the surrounding context points can also be considered. As a result, the model can allocate more attention resources to important areas, improving the accuracy of hypertension classification recognition.

#### 2.4.2. Model Parameters

After performing filtering and denoising on the data of all 30 volunteers using the preprocessing method described above, the PPG signal was sampled every 10 s and taken as one-dimensional data for training the model. Setting the learning rate is crucial in algorithm modeling. A low learning rate improves the reliability of the trained network, but it slows down the network parameter update speed and extends the training time. Conversely, a high learning rate may hinder network convergence and result in poor training effects. Additionally, a proper batch size setting can help the model determine the direction of gradient descent, which can improve the degree and speed of model optimization. To avoid overfitting during the model calculation process, the training process also introduced the dropout strategy. Table 4 shows the parameter settings of the LSTM model used in this research.

#### 2.4.3. Model Training

In this experiment, 70% of the data was used for training, 20% for testing, and 10% for validation. The LSTM-Attention recurrent network was trained, and the trained LSTM-Attention network was used to classify the test set. The model training was repeated multiple times using different random seeds to ensure the randomness of the data. Finally, the average of all predicted values was taken as the final result. The Keras deep learning framework [32] was used to establish and train the model.

#### 2.4.4. Performance Evaluation

The model’s performance was evaluated by calculating the probability distribution of the dataset being hypertensive, as well as the overall accuracy, precision, recall, and F1-score. The calculation method for accuracy is shown in Equation (12):

(12) $$\mathrm{Accuracy}=\frac{\mathrm{TP}+\mathrm{TN}}{\mathrm{TP}+\mathrm{TN}+\mathrm{FP}+\mathrm{FN}}$$

The calculation method for precision is shown in Equation (13):

(13) $$\mathrm{Precision}=\frac{\mathrm{TP}}{\mathrm{TP}+\mathrm{FP}}$$

The calculation method for recall is shown in Equation (14):

(14) $$\mathrm{Recall}=\frac{\mathrm{TP}}{\mathrm{TP}+\mathrm{FN}}$$

The calculation method for F1-score is shown in Equation (15):

(15) $$F1=\frac{2\times \mathrm{Precision}\times \mathrm{Recall}}{\mathrm{Precision}+\mathrm{Recall}}$$

where TP, TN, FP, and FN correspond to the relationships shown in Table 5.

During the training process, the loss function is the cross-entropy loss function, which is calculated using Equation (16):

(16) $$L\left(\theta \right)=-\frac{1}{N}\overset{N}{\underset{i=1}{\sum }}\overset{m}{\underset{j=1}{\sum }}\left(y_{i}\log\left(P\left(y_{i}={j|x}_{i}\right)\right)\right)$$

To further evaluate the performance of the proposed LSTM-Attention model for hypertension recognition, it was compared with other models, including LSTM (without attention mechanism), BiLSTM, SVM (Support Vector Machine), and KNN (K Nearest Neighbors), on the same dataset. For the LSTM and BiLSTM models, the training samples were the same as those used for the LSTM-Attention model. However, the traditional SVM and KNN models utilized a feature extraction method that involved manually extracting features before training. Specifically, 20-dimensional features were extracted in the time and frequency domains for each sample data, which consisted of PPG signals recorded every 10 s, as shown in Table 6.

With the increasing role of neural networks in the field of artificial intelligence, verifying their credibility has become a focus of attention, including their availability, reliability, robustness, and interpretability, among other factors [33]. In this experiment, a noise interference method was used to assess the robustness of the model, where unprocessed PPG test data was used to evaluate the model previously trained to verify its ability to resist interference. Furthermore, we used the data reservation method to assess the reliability of the LSTM-Attention model. We randomly selected and retained the data of one healthy volunteer and one patient with hypertension. The data of the remaining 28 volunteers were used as the training set to train the model, and the data of the two reserved volunteers were used as the test set to verify whether the model exhibited overfitting.

## 3. Results

**Figure 11:**
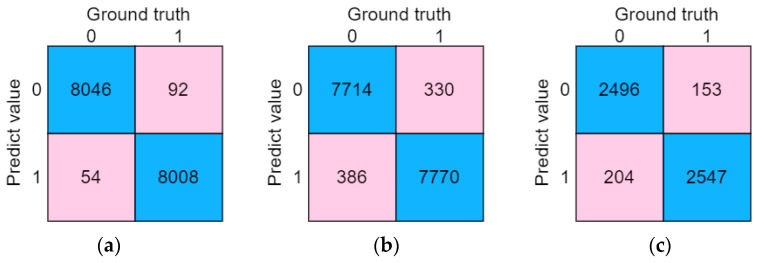
(**a**) Confusion matrix of the test set; (**b**) Confusion matrix of the robustness verification experiment; (**c**) Confusion matrix of the reliability verification experiment.

Based on the self-collected dataset, 70% of the pre-processed data was used to train the LSTM-Attention model. The confusion matrix on the test set is shown in Figure 11a. In the robustness verification experiment, a portion of unprocessed original PPG signals was used as the test set to verify the model, and the confusion matrix is shown in Figure 11b. In the reliability verification experiment, data from two volunteers were reserved for testing the model, and the confusion matrix is shown in Figure 11c.

**Figure 12:**
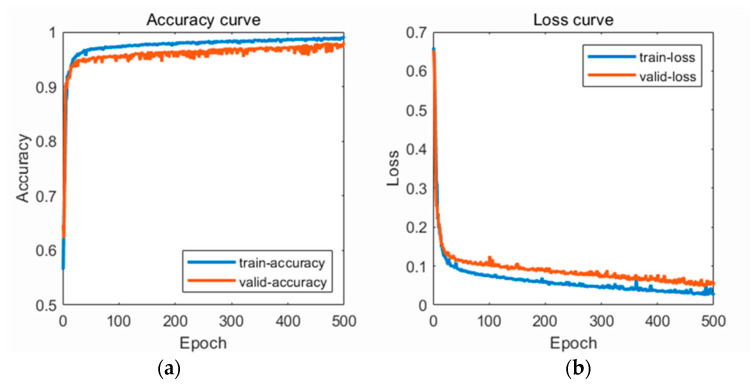
(**a**) Accuracy curve; (**b**) Loss curve.

Based on the confusion matrix, the recognition accuracy of the LSTM-Attention hypertension classification model was calculated as 99.1%, precision as 98.9%, recall as 99.3%, and F1-score as 99.1%. For the robustness verification, the classification accuracy of the unprocessed PPG data by the model was found to be 95.58%, precision as 95.89%, recall as 95.24%, and F1-score as 95.56%. For the reliability verification, the classification accuracy of the new data by the model was found to be 93.38%, precision as 94.22%, recall as 92.44%, and F1-score as 93.32%. The Accuracy and Loss curves for training and validation of the LSTM-Attention model are shown in Figure 12. As the number of iterations increases, the accuracy of the model gradually increases and reaches its highest value; the loss of the model gradually decreases and eventually converges to a stable value.

The classification results of different models on the same dataset are shown in Table 7. The LSTM model with the attention mechanism achieved the highest accuracy, reaching 99.1%, which is 3.8% higher than the accuracy of the regular LSTM model. The classification performance of the BiLSTM model was slightly higher than that of the LSTM model, reaching 95.8%. In contrast, the training and classification performance of SVM was the worst, with an accuracy of 90.4%, while KNN had a slightly higher accuracy than SVM, reaching 94.6%.

Table 8 displays the training time required for one epoch by different algorithm models. The LSTM model completes one epoch of training in 6.5 s, while the BiLSTM model takes 8.6 s. In comparison, the LSTM-Attention model requires 10.3 s for one epoch of training, indicating that the introduction of the attention mechanism increases the model’s complexity and requires a longer training time. Nevertheless, considering the improvement in the final accuracy, increasing the complexity of the model is necessary.

## 4. Discussion

The main findings of this study are as follows: (1) The PPG signal acquisition system designed and developed based on STM32 meets the experimental requirements and has the advantages of portability, low cost, and high accuracy. (2) The proposed LSTM-Attention hypertension recognition model has higher accuracy than the conventional RNN models. Compared with traditional machine learning methods, the proposed model avoids the cumbersome process of feature extraction and can better capture the correlation between PPG sequence information, enhance feature representation capability, and improve performance.

### 4.1. Performance of LSTM-Attention Model

The proposed LSTM-Attention model for hypertension recognition in this research achieved an outstanding accuracy of 99.1%, surpassing that of the conventional LSTM model at 95.3% and the BiLSTM model at 95.8%. The unidirectional LSTM model fell short of highlighting the connection between the current sampling point of the PPG signal and its historical and future information, making it unsuitable for long-term PPG signal recognition tasks. On the other hand, the LSTM model with an attention mechanism could allocate varying attention resources to sampling points of different importance levels, leading to a stronger feature expression ability. Furthermore, the LSTM-Attention model outperformed other models in terms of precision and recall evaluation. Nevertheless, as precision and recall mutually restrict each other, it is challenging to determine, objectively, which model is of higher quality. Hence, a more comprehensive evaluation index, the F1-score, is necessary for comparison. The F1-score of the LSTM-Attention model was 99.1%, which was still higher than the LSTM’s 95.2% and BiLSTM’s 95.7%. In the formal verification experiment, the classification accuracy of the LSTM-Attention model for the PPG data without preprocessing reached 95.58%. Although the accuracy slightly decreased when tested on noisy data, it still achieved a good recognition rate, indicating that the model had good robustness. When recognizing unfamiliar data, the accuracy rate reached 93.38%. Given the limited size of the dataset used in this experiment, such a fitting effect can be considered as meeting the requirements.

Traditional machine learning methods have been extensively studied in PPG [34,35,36,37], and this study also compares SVM and KNN classification models with the LSTM-Attention model. Unlike neural networks, which can directly train on raw signals, feature extraction is required before training these two models. In this study, PPG data was sampled at 10 s intervals, and 20 features in the time and frequency domains were extracted for each sample. The experimental results showed that the highest accuracy of SVM was 90.4%, and the highest accuracy of KNN was 94.6%, both of which were inferior to the LSTM-Attention model in evaluation metrics such as accuracy and precision. During the feature extraction process, it was found that the position of the reflected wave was difficult to locate or may disappear due to the influence of the elasticity of the vessel wall in hypertensive patients, leading to missing feature points and the need to discard such data, which reduced the overall data volume. Artificially extracted features have limited dimensions and make it difficult to discover correlations between features, which may overlook important features in identification and classification. The use of neural network methods can automatically obtain features and their correlations, avoiding the tedious work of manual feature extraction and enhancing the feature expression ability.

In terms of model efficiency, the LSTM model required 6.5 s for one round of training, while the BiLSTM model required 8.6 s. On the other hand, the LSTM model with an attention mechanism took 10.3 s for one round of training. When the sample size was small, there was little difference in the running time of the three models. However, as the sample size increased, the running time of the LSTM network with the attention mechanism significantly increased due to the attention mechanism’s considerable time consumption in identifying the focus and non-focus regions. This may slightly affect performance, but accuracy is crucial in hypertension recognition. Moreover, future hypertension recognition is likely to be more focused on personal applications with a limited sample size, where this algorithm’s advantages become more significant.

We compared our method with the results of previous studies, as shown in Table 9. These studies were focused on categorizing hypertension and non-hypertension using PPG signals. In the first study [38], 10 manually extracted features from PPG signals were used, and AdaBoost and KNN classifiers were employed as machine learning models. In the third [17] and fourth [18] studies, the scalogram and spectrogram of the PPG original waveform were used as input to the models, and the F1-scores obtained on the CNNs and BLSTM models reached 92.55% and 97.39%, respectively, which already outperformed traditional machine learning solutions. In contrast, the LSTM-Attention hypertension recognition model proposed in this study used raw data and neural networks to fully extract the deep features of PPG signals. The introduction of an attention mechanism strengthened the model’s feature expression ability in the long term, further improving its performance. The experimental results showed that our model achieved an accuracy of 99.1%, outperforming previous studies.

### 4.2. Prospect of Hypertension Identification

As mentioned in the introduction, PPG signals contain rich cardiovascular and cerebrovascular pathological information, which has led to an increasing number of studies using PPG for disease diagnosis. The integration of PPG technology into commonly used wearable devices, such as smart bracelets and watches, presents a promising opportunity for convenient and reliable early diagnosis of hypertension [39]. The PPG signal acquisition device designed in this study is lightweight and portable, as demonstrated in Table 2, with comparable accuracy to existing devices. The collected PPG data can be directly used for hypertension identification research. With the continuous advancement of microelectronic technologies, more advanced chips with greater computational power can be used in PPG devices, enabling the diagnosis of high blood pressure on wearable devices such as smart bracelets. This innovation could revolutionize traditional blood pressure measurements, allowing users to monitor their blood pressure health in real-time and receive alerts when their blood pressure is unhealthy, potentially preventing cardiovascular diseases. With the rapid development and widespread use of wearables, this research has the potential to benefit a larger population.

### 4.3. Limitations and Future Work

Several limitations may affect the experimental results. First, the number of volunteers recruited was not sufficient, and PPG data for all blood pressure values were not obtained. Although we considered the balance of age and gender, the limited amount of data may limit the generalizability of the model. Additionally, while PPG signals are easy to collect, they may have a poor signal-to-noise ratio and are susceptible to interference, which could have affected the data quality and affected the experimental results. Moreover, we only used collected red light PPG data for research, and more research is needed on the multidimensional and diversified fusion of data. To overcome these limitations, future work will involve recruiting more volunteers with different blood pressure values, monitoring the frequency drift of PPG signals to enhance the robustness of the dataset [16], and conducting multidimensional feature fusion research using PPG signals from multiple light sources. This will help extract hidden features in PPG signals from other wavelength light sources and enhance the reliability of the model.

## 5. Conclusions

This research designed a PPG signal acquisition device for hypertension recognition and proposed an LSTM-Attention hypertension recognition model based on the attention mechanism. The introduction of an attention mechanism improved the feature expression ability of the model, thereby improving the recognition accuracy. Experimental results showed that the accuracy of the LSTM-Attention hypertension recognition model reached 99.1%. Compared with other machine learning evaluation models, this model demonstrated excellent performance, ranking first in precision, accuracy, and F1-score indicators. Although there was some sacrifice in model complexity, the overall performance was improved. In summary, the proposed LSTM-Attention hypertension recognition model has significant advantages and has the potential to be used for hypertension assessment in home and community environments. Future work will involve using multi-source PPG signals to train a hypertension recognition model with stronger feature expression ability.

## Figures and Tables

**Table 1:** Parameters of PPG acquisition equipment.

Parameter	Value
Dimension (mm)	45 × 38 × 20
LED wavelength (nm)	527/660/880
Type of light source	Green/Red/Infrared light
PPG sampling form	Light reflection
LED supply voltage (V)	3.3
Working current (mA)	1.5
Sampling rate (Hz)	100
Battery capacity (mAh)	400

**Table 2:** Comparison of heart rate with DB12 blood oxygen monitor.

No. of Volunteer	Experimental Data/bpm	DB12/bpm	Error
	89	91	2%
1	87	87	0%
	90	89	1%
	83	83	0%
2	85	86	1%
	81	82	1%
	78	80	2%
3	79	80	1%
	76	76	0%

**Table 3:** Comparison of basic data between two groups of volunteers.

Group	No. of Males (%)	Age	BMI/(kg/m^2^)	SBP/(mmHg)
Healthy	8 (53.3)	54.7 ± 5.9	21.6 ± 2.7	115.4 ± 8.9
Hypertension	8 (53.3)	55.3 ± 5.7	25.5 ± 2.9	143.6 ± 7.5

Age, BMI, and SBP are represented by Average ± Standard Deviation.

**Table 4:** Parameters of LSTM model.

Parameter	Value
Activation function	ReLU
Classifier	SoftMax
Learn-rate	0.001
LSTM-layers	5
Batch-size	256
Num-epochs	500
Dropout	0.5

**Table 5:** Mapping between TP, TN, FP, and FN.

Confusion Matrix		Ground Truth	
		Positive	Negative
Predicted value	Positive	TP	FP
	Negative	FN	TN

**Table 6:** Time domain and frequency domain characteristics of PPG.

Characteristic	Definition
x	The amplitude of the dominant wave
y	The amplitude of the dicrotic wave
z	The amplitude of the dicrotic notch
y/x	The ratio of y to x
x−y/x	The ratio of x − y to x
$t_{\mathrm{pp}}$	The duration between the main wave peaks of two adjacent PPG waveforms
$t_{1}$	The duration from trough to the peak of a single PPG waveform
$t_{2}$	The duration from trough to the dicrotic notch of a single PPG waveform
$t_{3}$	The duration from trough to the dicrotic wave peak of a single PPG waveform
$t_{4}$	The duration between the trough of two adjacent PPG waveforms
∆T	The duration from principal wave peaks to dicrotic wave peak
$t_{1}/t_{\mathrm{pp}}$	The ratio of $t_{1}$ to $t_{\mathrm{pp}}$
$t_{2}/t_{\mathrm{pp}}$	The ratio of $t_{2}$ to $t_{\mathrm{pp}}$
$t_{3}/t_{\mathrm{pp}}$	The ratio of $t_{3}$ to $t_{\mathrm{pp}}$
$∆T/t_{\mathrm{pp}}$	The ratio of ∆T to $t_{\mathrm{pp}}$
Fre1	The frequency of Peak1 after the Fourier transform
Fre2	The frequency of Peak2 after the Fourier transform
Fre3	The frequency of Peak3 after the Fourier transform
$e_{1}$	Shannon Entropy
$e_{2}$	Information Entropy

**Table 7:** Classification results of different models on the dataset.

Model	Accuracy	Precision	Recall	F1-Score
LSTM-Attention	0.991	0.989	0.993	0.991
LSTM	0.953	0.971	0.934	0.952
BiLSTM	0.958	0.975	0.940	0.957
SVM	0.904	0.917	0.938	0.927
KNN	0.946	0.936	0.984	0.959

**Table 8:** Training time for one epoch of different models.

Model	Time/s
LSTM-Attention	10.3
LSTM	6.5
BiLSTM	8.6
SVM	NA
KNN	NA

**Table 9:** Classification performance comparison.

Model	Feature Extraction	Database	Classifier	F1-Score
PPG features [38]	10 PPG features	121 subjects (MIMIC database)	AdaBoost	80.11%
PPG features [38]	10 PPG features	121 subjects (MIMIC database)	KNN	86.94%
Raw PPG signal [17]	Short-time Fourier transform (spectrogram)	219 subjects (Figshare database)	BLSTM with time-frequency analysis	97.39%
Raw PPG signal [18]	Continuous wavelet transform (scalogram)	219 subjects (Figshare database)	CNNs	92.55%
Raw PPG signal (current study)	Raw PPG signal after preprocessing	30 subjects (Self-collecting database)	LSTM-Attention	99.10%

## Data Availability

Data is unavailable due to privacy and ethical restrictions.
